# The glymphatic system as a therapeutic target: TMS-induced modulation in older adults

**DOI:** 10.3389/fnagi.2025.1597311

**Published:** 2025-07-17

**Authors:** Mark H. Sundman, Yilin Liu, Nan-kuei Chen, Ying-hui Chou

**Affiliations:** ^1^Brain Imaging and TMS Laboratory, Department of Psychology, University of Arizona, Tucson, AZ, United States; ^2^Department of Biomedical Engineering, University of Arizona, Tucson, AZ, United States; ^3^Evelyn F McKnight Brain Institute, Arizona Center on Aging, and BIO5 Institute, University of Arizona, Tucson, AZ, United States

**Keywords:** transcranial magnetic stimulation, mild cognitive impairment, glymphatic system, APOE, DTI-ALPS

## Abstract

While repetitive transcranial magnetic stimulation (rTMS) is a promising neuromodulatory intervention for cognitive impairment, its effects on the glymphatic system remain unexplored in clinical populations. Deficient glymphatic clearance has emerged as a central feature of neurodegenerative disease, which can now be assessed with specialized diffusion magnetic resonance imaging techniques. This study examines changes in the diffusion tensor imaging analysis along the perivascular space (DTI-ALPS) index following theta-burst stimulation (TBS) in older adults with mild cognitive impairment (MCI). DTI-ALPS is an MRI-based measure that reflects the efficiency of the brain’s glymphatic waste removal system, as it quantifies how easily water molecules move along the perivascular spaces where waste is cleared. Participants underwent ten consecutive days of continuous TBS, intermittent TBS, and sham TBS, with DTI-ALPS measurements acquired before and after each intervention. Our sham-controlled findings reveal the capacity for TBS interventions to modulate glymphatic function and highlight a significant APOE ε4 effect. Specifically, ε4 carriers exhibited a lower baseline DTI-ALPS index (*p* < 0.05, Cohen’s d = 0.610), suggesting reduced glymphatic function, which was selectively responsive to TBS interventions (*p* < 0.005, Cohen’s d = 1.71). Further, within this subgroup, TBS-induced increases in glymphatic function correlated with memory improvements (*r* = 0.42–0.46, *p* < 0.05). These results provide novel evidence that TBS can modulate glymphatic function in humans and raise interesting questions about the relevance of APOE status. Further research is needed to elucidate the mechanisms underlying these effects and their therapeutic implications.

## Introduction

All biological processes rely on energy production, and the metabolic activity required to produce this energy inevitably produces waste products. In peripheral tissues, the well-characterized lymphatic system efficiently clears the associated byproducts to maintain fluid homeostasis and mitigate potential harm (Oliver et al., 2020). The central nervous system (CNS), however, has long been considered devoid of histologically distinct lymphatic structures, presenting a challenge in understanding how the brain, one of the most metabolically active organs, manages the clearance of potentially harmful waste products (Mehta and Mehta, 2024). This biological contradiction persisted until 2012, when experiments began to characterize the glymphatic system, a glia-dependent pathway for waste clearance in the brain, offering critical insight into CNS homeostasis (Iliff et al., 2012; Iliff et al., 2013).

While this novel system is not yet fully understood, the glymphatic system broadly depends on the exchange of cerebrospinal fluid (CSF) and interstitial fluid (ISF), driven by the convective inflow of CSF into perivascular spaces. From the perivascular space, subsequent fluid transport through the brain parenchyma is facilitated by astrocytic endfeet, which enwrap the cerebral vascular tree and are enriched with a highly polarized distribution of aquaporin-4 (AQP4) water channels (Mestre et al., 2018; Salman et al., 2021). The waste-laden solute then drains through meningeal lymphatic vessels (mLVs) before reaching the peripheral lymphatic system near the base of the skull (Jessen et al., 2015; Li et al., 2022). As individuals age, the glymphatic system becomes increasingly susceptible to functional decline (Zhou et al., 2020). For example, age-or disease-related changes in astrocytic morphology can profoundly disrupt the clearance of potentially harmful metabolic byproducts like *β*-amyloid (Aβ) (Das et al., 2024; Palmer and Ousman, 2018).

Indeed, deficient glymphatic clearance has been implicated in the pathogenesis of numerous aging processes and disease states, including Alzheimer’s disease (AD). Aβ accumulation, a hallmark of AD, underscores the critical link between brain energy demand, metabolic clearance, and disease. In healthy systems, Aβ, a byproduct of normal neuronal activity, is cleared from the brain via astrocytic-dependent glymphatic processes (Carlstrom et al., 2022; Chen et al., 2017). The clearance of Aβ and other byproducts typically follows a diurnal pattern, peaking at night during slow-wave sleep (SWS), when heightened neural synchrony enhances fluid exchange in perivascular spaces (Reddy and van der Werf, 2020; Xie et al., 2013; Shokri-Kojori et al., 2018). With deficient glymphatic clearance from the CNS, this toxic protein can accumulate in neuronal tissue, exacerbating neurodegenerative processes and accelerating disease progression. In Alzheimer’s Disease and Related Dementia (ADRD), aberrant accumulation of Aβ occurs 15 years before the onset of clinically evident cognitive impairment (Dubois et al., 2016).

Mounting evidence implicates glymphatic failure as a common pathway in the multifaceted pathophysiological cascade of ADRD. Although this novel system is not yet fully understood, numerous aspects of glymphatic functionality are intertwined with the disease’s pathophysiology (Nedergaard and Goldman, 2020). These include astrocytic dysfunction, disrupted sleep architecture, impaired arterial pulsatility, reduced synchronous neural activity, and shrinkage of meningeal lymphatic vessels (mLVs) (Zhang et al., 2019; Rajna et al., 2021; Rodriguez-Arellano et al., 2016; Mentis et al., 2021). Collectively, these factors disrupt CSF-ISF fluid exchange and subsequent transport, diminishing the glymphatic clearance of metabolic byproducts such as Aβ (Rasmussen et al., 2018).

Notably, the physiological pathways that support glymphatic clearance and facilitate the removal of pathogenic proteins are closely intertwined with the function of Apolipoprotein E (APOE), the strongest known genetic risk factor for sporadic AD. Though carried by only ~15% of individuals, the ε4 allele is implicated in up to 50% of AD cases and is associated with both accelerated Aβ accumulation and earlier disease onset (Mann et al., 1997; Ashford, 2004; Corder et al., 1993). A key contributor to APOE’s multifaceted impact on AD risk is the impaired clearance of Aβ and tau conferred by the ε4 allele (Eisenbaum et al., 2024; Hudry et al., 2013; Castellano et al., 2011; Simonovitch et al., 2016; Liu et al., 2017; Deane et al., 2008). Relatedly, glial cells—the namesake of the glymphatic system—have emerged as central mediators of ε4-related pathology. Astrocytes, which facilitate glymphatic function via AQP4 water channels, may be particularly susceptible to ε4-induced disruption (Fernandez et al., 2019). This was elegantly demonstrated by a recent experiment in which sleep deprivation selectively amplified AD pathology in mice expressing human APOE4 (Wang et al., 2023a). This ε4-specific vulnerability was linked to glial changes, including reduced AQP4 expression and a loss of its polarized localization on astrocytic endfeet. These findings highlight the potential relevance of APOE for glymphatic dysfunction and AD risk, particularly in response to common physiologic stressors such as sleep disruption.

The emergence of glymphatic function as a central feature of ADRD has spurred research efforts to non-invasively evaluate the system’s integrity (Taoka and Naganawa, 2020). The current gold standard entails an invasive approach through which glymphatic drainage is assessed with contrasted magnetic resonance imaging (MRI) following intrathecal administration of a gadolinium-based contrast agent (Eide and Ringstad, 2015). This invasive approach, however, requires a lumbar puncture and multiple MRI scans across fixed intervals, limiting its clinical feasibility. Alternative non-invasive and contrast-free MRI techniques have recently emerged, demonstrating high validity when directly compared to this gold standard (Zhang et al., 2021). One such approach is a diffusion MRI technique termed diffusion tensor image analysis along the perivascular space (DTI-ALPS) (Taoka et al., 2017). This method yields an ALPS-index that may offer some insight into glymphatic drainage (i.e., a higher ALPS-index could suggest more glymphatic flow), and is bolstered by evidence of high reproducibility (Han et al., 2023; Taoka et al., 2022). Although DTI-ALPS should not be misconstrued as a direct measure of whole-brain glymphatic function, the ALPS index provides a useful proxy for localized perivascular fluid dynamics and offers meaningful insights into this physiological system, despite its methodological limitations.

Since its introduction, the DTI-ALPS approach has been widely adopted as a method for assessing glymphatic function across the continuum of AD (Wang et al., 2023b; Park et al., 2023; Hsu et al., 2023; Chang et al., 2023; Okazawa et al., 2024; Kim et al., 2024; Huang et al., 2024; Hong et al., 2024; Zhang et al., 2024; Steward et al., 2021; Kamagata et al., 2022; Li et al., 2024). From this work, multimodal neuroimaging studies employing positron emission tomography (PET) and diffusion MRI report a negative association between whole-brain Aβ burden and the ALPS index (Hong et al., 2024; Hsu et al., 2023). Additionally, consistent reports show that the ALPS index correlates significantly with worse cognitive performance across multiple domains (Hong et al., 2024; Zhang et al., 2024; Kamagata et al., 2022; Hsu et al., 2023). A recent longitudinal study offers compelling evidence that DTI-ALPS metrics can predict the progression of AD, suggesting that glymphatic dysfunction may precede and accelerate the development of hallmark Aβ pathology (Huang et al., 2024). Specifically, the study found that a lower ALPS index was significantly associated with (1) increased PET-detected Aβ burden, (2) accelerated atrophy in AD-signature brain regions, (3) a higher risk of transitioning to Aβ-positive status, (4) faster clinical progression, and (5) more rapid cognitive decline (Huang et al., 2024).

These findings present a compelling rationale for novel therapeutic strategies targeting the glymphatic system (Gao et al., 2023). The well-documented link between sleep architecture and glymphatic function underscores the plasticity of this system, as both human and animal data demonstrate enhanced glymphatic activity during non-REM slow-wave sleep (SWS) (Hablitz et al., 2019; Fultz et al., 2019). More recently, elegant research isolated large-scale, synchronized neural activity as a key driver of enhanced glymphatic flux during SWS (Jiang-Xie et al., 2024; Murdock et al., 2024). In one experiment, multisensory gamma stimulation enhanced glymphatic clearance in an AD mouse model by increasing arterial pulsatility, promoting AQP4 polarization in astrocytic endfeet, and dilating meningeal lymphatic vessels (mLVs) (Murdock et al., 2024). Parallel research demonstrated that synchronized neuronal firing at multiple frequencies enhances glymphatic flux, providing novel insights into the role of large ion gradients in this process (Jiang-Xie et al., 2024). Summarizing their findings, the authors remarked, “*In essence, neurons that fire together ‘shower’ together*” (Jiang-Xie et al., 2024).

While therapeutic interventions that mitigate sleep disturbances offer a relatively simple approach to enhance glymphatic function (Saito et al., 2023), more novel interventions—pharmacological or otherwise—warrant exploration. For example, early evidence from animal models highlights repetitive transcranial magnetic stimulation (rTMS), a non-invasive brain stimulation technique, as a promising candidate for therapeutically targeting the glymphatic system (Wu et al., 2022; Lin et al., 2021; Liu Y. et al., 2023; Li et al., 2020). In AD mouse models, rTMS has been shown to enhance glymphatic fluid transport, thereby reducing Aβ burden. Mechanistically, these effects are reportedly mediated by (1) reduced astrocytic reactivity (Lin et al., 2021), (2) improved AQP4 polarization on astrocytic endfeet (Wu et al., 2022), and (3) mLV dilation mediated by vascular endothelial growth factor C (VEGF-C) (Li et al., 2020). Though the cellular mechanisms that underpin rTMS therapies are not fully understood, extensive evidence supports its capacity to modulate glial function, promote angiogenesis, and support neurovascular remodeling (Ferreira et al., 2024; Hong et al., 2020; Qian et al., 2024; Cirillo et al., 2017; Zong et al., 2020; Ljubisavljevic et al., 2015; Zong et al., 2022). For example, findings across different animal models include TBS-induced restoration of AQP4 expression and its polarized localization on astrocytic endfeet, further reinforcing its therapeutic potential for enhancing glymphatic function (Lin et al., 2024; Wu et al., 2022).

Building on this mounting preclinical evidence, we sought to investigate whether rTMS similarly influences the DTI-ALPS index which may represent glymphatic function in humans. In this study, we assessed changes in DTI-ALPS following rTMS therapy in older adults with mild cognitive impairment (MCI). For all participants, we compared DTI-ALPS metrics at baseline and again following ten consecutive days of three distinct rTMS interventions: continuous theta-burst stimulation (cTBS), intermittent theta-burst stimulation (iTBS), and sham-TBS. TBS has emerged as a neuromodulatory approach with comparable, if not superior, efficacy to conventional rTMS and offers key advantages in efficiency, reduced treatment time, and potential cost savings (Tao et al., 2024; Chung et al., 2015). To our knowledge, this is the first report of DTI-ALPS following rTMS in human participants.

## Methods

### Experimental design

Thirty-six right-handed individuals with mild cognitive impairment (MCI) participated in this study (age: 66.1 ± 7.45 yrs.; females: 28, mean years of education: 16.38 ± 2.21 yrs.). Only right-handed participants were included to reduce variability related to hemispheric lateralization, particularly given the left-hemispheric targeting used in this study. The dataset consisted of 36 right-handed individuals determined to have mild cognitive impairment (MCI; age: 66.1 ± 7.45 years old; females: 28, education: 16.375 ± 2.21 years). MCI was classified according to the revised Mayo Clinic Criteria and supported by the Jak/Bondi neuropsychological actuarial approach. The Mayo criteria include self-or informant-reported cognitive concerns, objective cognitive impairment, preserved functional independence, and the absence of dementia. Objective cognitive impairment and MCI subtypes were determined using the Jak/Bondi criteria (Bondi et al., 2014), according to age-, sex-and education-adjusted scores from the National Alzheimer’s Coordinating Center Uniform Data Set Neuropsychological Battery, Version 3 (UDSNB-3).

**Figure 1:**
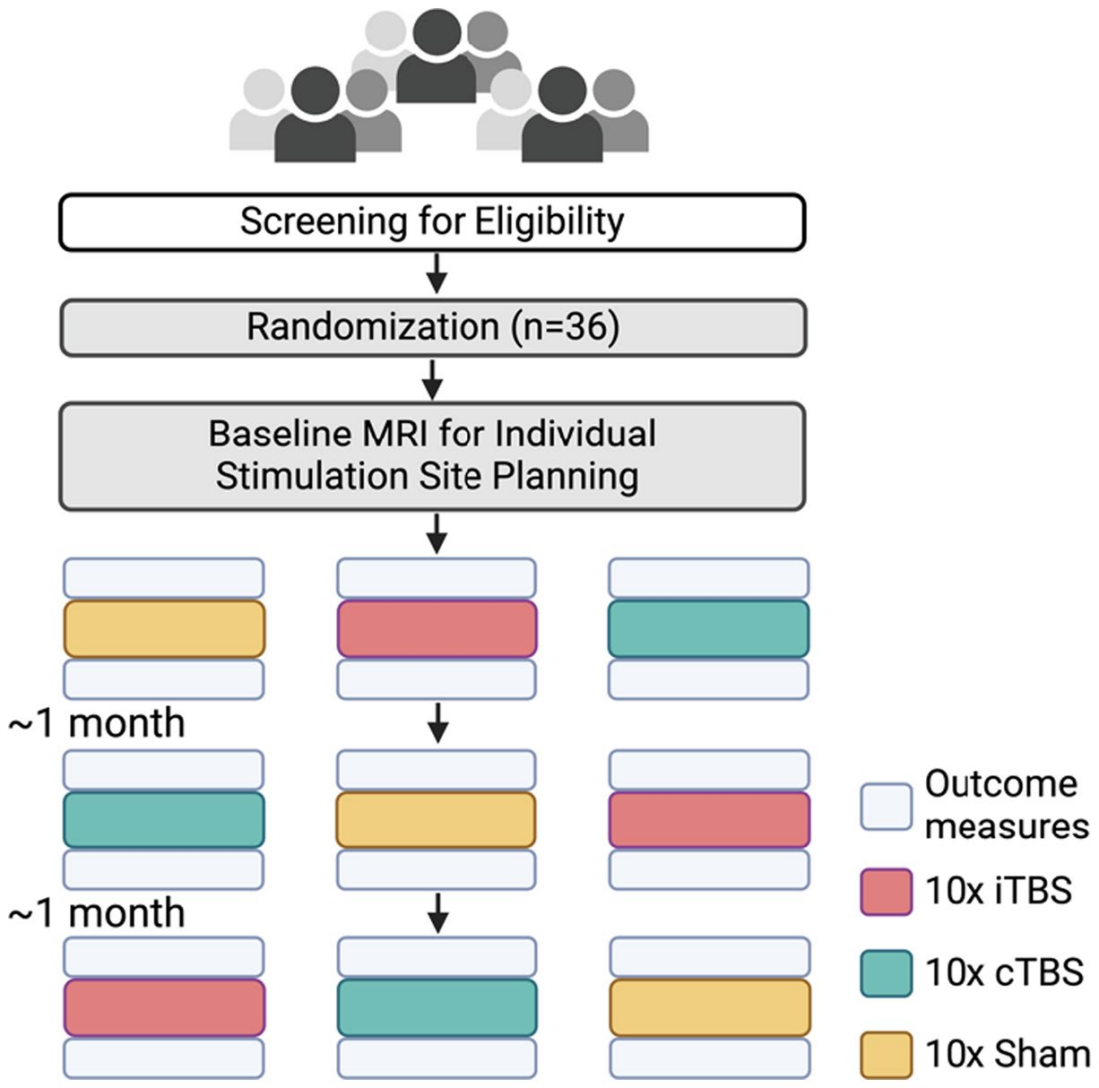
Experimental design. Participants (*n* = 36) were screened for eligibility and randomized into three groups: intermittent theta-burst stimulation (iTBS), continuous theta-burst stimulation (cTBS), and Sham stimulation (10 sessions each). Baseline diffusion MRI was performed to guide individual stimulation site planning. Outcome measurements include the face-name association memory performance and DTI-ALPS index. Outcome measurements were taken before and after each TBS block.

As shown in Figure 1, each participant underwent three TBS conditions in a randomized order: intermittent theta burst stimulation (iTBS), continuous theta burst stimulation (cTBS), and sham TBS. TBS is a patterned form of TMS (Huang et al., 2005), which involves delivering rapid bursts of TMS pulses at the theta-band frequency (Larson et al., 1986). Participants completed ten TBS sessions on consecutive weekdays for each condition, with a one-month washout interval between each condition to prevent potential carry-over effects. The full study protocol spanned approximately 6 months per participant, including stimulation sessions and washout intervals. TBS was delivered to a personalized stimulation site within the left parietal cortex, determined using a DTI-guided voxel-based strategy previously described (Liu et al., 2024). The TMS coil orientation was optimized with the SimNIBS toolbox, which models the induced electric fields based on each individual’s structural MRI data (Gomez et al., 2021).

Repeated outcome measures were collected at six time points: once the day before the first TBS session and again immediately after the final TBS session for each TBS condition. At each evaluation, data was collected to assess the DTI-ALPS and associative memory performance with the face-name associative memory exam (FNAME). The FNAME task included six distinct and parallel versions, which were randomized and administered pre-and post-TBS for each protocol condition in a counterbalanced fashion. These versions were previously evaluated in-house and demonstrated high test–retest reliability (ICC > 0.9) with no evidence of practice effects across repeated administrations. The FNAME provided measures of accuracy and sensitivity (d1), with additional details included in the statistical analysis section. Distinct versions of the FNAME task were utilized at each time point to minimize practice effects. Further details of the FNAME are provided in Supplementary Item S5.

### Theta burst stimulation (TBS)

Two active TBS protocols were implemented: iTBS and cTBS. iTBS was composed of 600 biphasic pulses, patterned in 3-pulse bursts at 50 Hz, repeated at 5 Hz, and delivered as intermittent trains of 2 s each, with an 8-s intertrain interval (Huang et al., 2005). cTBS consisted of 600 continuous stimuli without intertrain intervals. For the sham TBS condition, we used a sham coil specifically designed for blinded clinical trials. The order of all three stimulation blocks was randomized for each participant.

### DTI-ALPS index calculation

Prior to DTI-ALPS analysis, diffusion-weighted images underwent a series of preprocessing steps to improve data quality and reduce artifacts. The pipeline began with MP-PCA-based denoising to suppress random noise while preserving anatomical detail, followed by Gibbs ringing correction using the method of sub-voxel shifts. These steps were performed using MRtrix3. The preprocessed data then underwent susceptibility distortion correction using reversed phase-encoding b0 images, followed by eddy current and motion correction to account for participant movement and gradient-induced distortions. Motion outliers were identified using FSL’s eddy QC tools, and datasets with excessive motion (e.g., >3 mm absolute displacement or >20% outlier slices) were excluded from further analysis. We then employed a previously established DTI-ALPS processing pipeline implemented using the FMRIB Software Library (FSL). The FSL pipeline consisted of artifact corrections, including MP-PCA denoising and Gibbs unringing, applied via MRtrix3 commands. Additional corrections for susceptibility-induced distortions, eddy currents, and movements were performed with standard FSL commands.

Template co-registration was performed using the JHU-ICBM-FA-1 mm template. Regions of Interest (ROIs) were automatically defined as 5 mm spheres and placed bilaterally in the superior corona radiata (SCR), a projection fiber, and the superior longitudinal fasciculus (SLF), an association fiber, using the JHU-ICBM-FA template (Hua et al., 2008). The DTI-ALPS index, defined as the mean of the bilateral DTI-ALPS indexes, is a ratio of mean diffusivity in different directions. Specifically, it is calculated as the ratio of mean x-axis diffusivity in the projection (D_xxproj_) and association (D_xxassoc_) fiber areas to the mean y-axis diffusivity in projection fiber areas (D_yyproj_) and z-axis diffusivity association fiber areas (D_zzassoc_). We computed DTI-ALPS indexes for the left, right, and bilateral hemispheres before and after each TBS protocol.

### APOE genotypes

For the analysis of APOE ε4 allele, DNA samples were obtained via an oral swab kit and dispatched to the Genetics Core for genotyping analysis of selected genes. The APOE genotyping, focusing on SNP rs429358 and SNP rs7412, was performed using TaqMan® Assays (Applied Biosystems, Foster City, CA, USA) and TaqMan™ Fast Advanced Master Mix (Thermo Fisher Scientific, Waltham, MA, USA) on an Applied Biosystems 7,300 Real-Time PCR System according to the manufacturer’s protocol. Genotype determination was conducted utilizing SDS v1.4 software from Applied Biosystems.

### Statistical analysis

We first evaluated the baseline differences in Sham-controlled DTI-ALPS index between APOE ε4 carriers and non-carriers at the pre-intervention timepoint with an independent two-sample Welch’s t-test. This approach was chosen due to the unequal sample sizes between the APOE ε4 carrier (*n* = 13) and non-carrier (*n* = 23) groups, as it does not assume homogeneity of variance. To isolate the effect of active stimulation from non-specific changes such as test–retest variability or scanner drift, we computed Sham-controlled DTI-ALPS indices by subtracting the corresponding Sham condition values from each active condition. This subtraction controls for non-stimulation-related influences and allows for a more accurate assessment of the net effect of active TBS. The derived indices were calculated as follows:

Sham-controlled iTBS pre = Pre-iTBS DTI-ALPS index—Pre-Sham DTI-ALPS index.
Sham-controlled cTBS pre = Pre-cTBS DTI-ALPS index—Pre-Sham DTI-ALPS index.
Sham-controlled iTBS post = Post-iTBS DTI-ALPS index—Post-Sham DTI-ALPS index.
Sham-controlled cTBS post = Post-cTBS DTI-ALPS index—Post-Sham DTI-ALPS index.

We further used linear mixed-effects (LME) models with repeated measures to analyze the relationship between DTI-ALPS values (left, right, and bilateral) and the effects of TIME (pre-TBS vs. post-TBS), APOE ε4 group (carrier vs. non-carrier), and Protocol (Sham-controlled iTBS vs. Sham-controlled cTBS). In the LME model, TIME-pre served as the reference for comparisons with TIME-post, APOE ε4 non-carriers were the reference group for APOE ε4 carriers. Sex was included as a covariate to account for potential sex-related variance. To account for this baseline group difference, the LME analysis incorporated subject-specific variability and controls for potential pre-existing disparities in the DTI-ALPS values.

We included interaction terms to explore the combined effects of Time × APOE ε4, Time × Protocol, and APOE ε4 × Protocol, as well as the three-way interaction of Time × APOE ε4 × Protocol. A random intercept was added for each participant to account for within-subject correlations. For any significant interaction effects observed in the model, *post hoc* analyses were performed using pairwise comparisons with Bonferroni corrections. TBS protocol (iTBS vs. cTBS) and its interactions with Time and APOE ε4 status were modeled in the linear mixed-effects analysis. The absence of significant interaction effects indicated no protocol-specific influence on the DTI-ALPS index, supporting the decision to combine the active protocols for subsequent analysis.

As a secondary analysis, we examined how changes in FNAME task performance were related to changes in the DTI-ALPS index, with results stratified by APOE ε4 status. FNAME outcome measures include accuracy (total correct responses over all trials) and a sensitivity measure, d1, calculated as d1 = Z(Hit rate) – Z(False alarm rate) (Carr et al., 2017). Changes in FNAME performance were computed as the difference between post-and pre-TBS scores for each active TBS condition, relative to the corresponding difference in the sham condition. For example, the change in FNAME performance following iTBS was calculated as: Δi TBS-accuracy = (Post-iTBS accuracy−Pre-iTBS accuracy) − (Post-Sham accuracy−Pre-Sham accuracy).

All statistical analyses were conducted using R version 3.5.1. Statistical significance was determined at a threshold of *p* < 0.05. Where applicable, multiple comparisons were corrected using the Bonferroni method.

## Results

Of the 36 participants with MCI included in the study, 13 individuals were identified as APOE ε4 carriers (Age: 64.30 yrs., Female: 10, Education: 15.96 yrs) and 23 as non-carriers (Age: 67.13 yrs., Female: 18, Education: 16.61 yrs) (see Table 1).

**Table 1:** Demographic and clinical characteristics.

Characteristic	Total (*N* = 36)	APOE ε4 Carriers (*n* = 13)	Non-Carriers (*n* = 23)
Age, years (SD)	66.11 (7.4)	67.13 (8.9)	65.50 (5.5)
Female sex, *n* (%)	28 (77%)	10 (77%)	18 (78%)
Education, years (SD)	16.37 (2.2)	16.61 (2.2)	15.96 (2.1)
MoCA, mean (SD)	24.69 (2.2)	24.65 (1.8)	24.76 (2.1)
Amnestic MCI, *n* (%)	19 (53%)	9 (69%)	10 (43%)

At baseline, the independent Welch’s t-test revealed a statistically significant difference between the APOE ε4 carriers and non-carriers with Sham-controlled bilateral DTI-ALPS index, *t*(25.54) = 2.4948, p = 0.0158, with a medium effect size (Cohen’s d = 0.61). Specifically, APOE ε4 carriers (M = −0.0199, SD = 0.0436) exhibited significantly lower baseline bilateral DTI-ALPS index compared to non-carriers (M = 0.0070, SD = 0.0447). A significant difference was also observed in the right DTI-ALPS index, with APOE ε4 carriers (M = −0.0224, SD = 0.0386) showing significantly lower values than non-carriers (M = 0.0119, SD = 0.0481), *t*(29.83) = 3.31, *p* = 0.0016. This effect was associated with a large effect size (Cohen’s d = 0.79). No significant difference was found in the left DTI-ALPS index between APOE ε4 carriers (M = −0.0199, SD = 0.0436) and non-carriers (M = 0.0070, SD = 0.0447), *t*(24.82) = 1.3246, *p* = 0.1911. This suggests that APOE ε4 carriers may have reduced glymphatic function at baseline, as indicated by the DTI-ALP index, before any TBS intervention.

**Figure 2:**
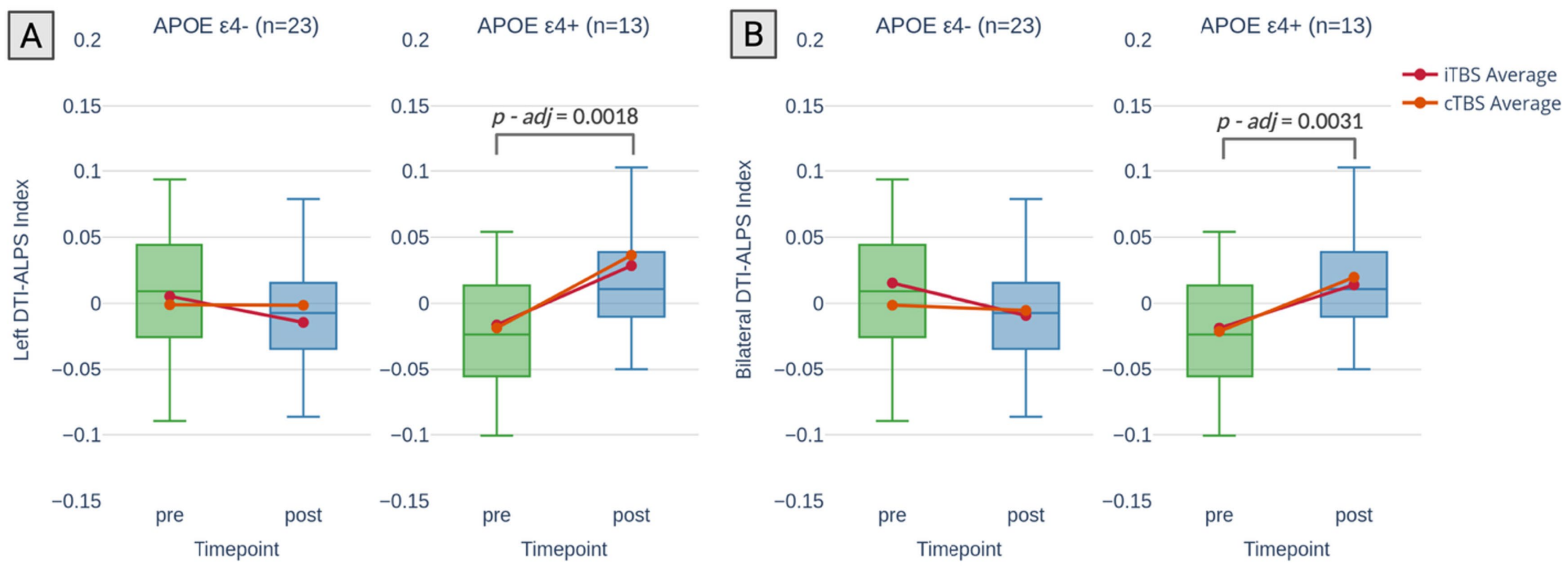
Changes in DTI-ALPS index by APOE ε4 status. Changes in DTI-ALPS index stratified by APOE ε4 status (non-carriers: APOE ε4-; carriers: APOE ε4+) across timepoints (pre-and post-TBS). **(A)** Left DTI-ALPS index: A significant increase in index was observed for APOE ε4 carriers following active TBS, with no significant change in non-carriers. **(B)** Bilateral DTI-ALPS index: A significant increase in index was also observed for APOE ε4 carriers, while no significant change was noted in non-carriers. The *p*-values displayed in the figure are corrected for multiple comparisons following *post hoc* analysis.

Table 2 presents the sham-controlled changes in DTI-ALPS index following a TBS intervention across different APOE groups. A significant TIME x APOE ε4 status interaction was observed for bilateral DTI-ALPS (*p* = 0.002), left DTI-ALPS (*p* = 0.007) and right DTI-ALPS (*p* = 0.015), indicating that the increase in DTI-ALPS scores after active TBS was moderated by APOE ε4 status. This Time x APOE4 interaction corresponded to large standardized effect sizes for bilateral (Cohen’s d = 1.71), left (Cohen’s d = 1.50), and right (Cohen’s d = 1.30) DTI-ALPS, reflecting a stronger pre–post increase in ε4 carriers compared to non-carriers. *Post hoc* sensitivity analysis revealed a minimum detectable effect size of *f* = 0.48 with 80% power at *α* = 0.05. Given the approximate relationship between Cohen’s d and f (i.e., f ≈ d/2 when comparing two groups), the observed interactions (with d values ranging from 1.30 to 1.71) correspond to *f* values of approximately 0.65 to 0.86—well above the detectable threshold. The results demonstrate large effect sizes and suggest that the study was sufficiently powered to detect the key APOE-related differences in DTI-ALPS. Additionally, no significant three-way interaction between TIME, APOE ε4 status, and TBS protocol was found, suggesting that the above-mentioned TIME x APOE ε4 status interaction was independent of whether the participants received iTBS or cTBS. Therefore, iTBS and cTBS were combined in the subsequent analysis. In the *post hoc* analysis, a significant increase in the left DTI-ALPS was observed among APOE ε4 carriers following active TBS (*t* = 3.740, *p-adj* = 0.0018; Figure 2A). Similarly, bilateral DTI-ALPS scores also increased significantly in this group (*t* = 3.591, *p-adj* = 0.0031; Figure 2B). Multiple comparisons were corrected using the Bonferroni method for all post hoc tests. While the effect in the right DTI-ALPS showed an uncorrected positive trend (*t* = 2.087, *p* = 0.0411), it did not reach statistical significance after correction (*p-adj* = 0.1649).

**Table 2:** Linear mixed effect model analysis: protocol, APOE ε4 status, time on sham-controlled DTI-ALPS index.

Outcomes	Fixed effects	Estimate	Std. Error	t value	95% confidence interval		*P* value
					Lower	Upper	
Bilateral DTI-ALPS	Time	−0.025	0.011	−2.256	−0.046	−0.003	0.026 *
	Protocol	−0.017	0.011	−1.540	−0.038	0.005	0.127
	Sex	0.014	0.011	1.246	−0.008	0.035	0.222
	APOE ε4 status	−0.034	0.015	−2.329	−0.063	−0.005	0.022 *
	Time * Protocol	0.021	0.015	1.337	−0.010	0.051	0.184
	Timepoint * APOE ε4 status	0.057	0.018	3.156	0.022	0.093	0.002 **
	Protocol * APOE ε4 status	0.014	0.018	0.784	−0.021	0.050	0.435
	Time * Protocol * APOE ε4 status	−0.012	0.026	−0.479	−0.062	0.038	0.633
Left DTI-ALPS	Time	−0.020	0.014	−1.395	−0.048	0.008	0.166
	Protocol	−0.006	0.014	−0.453	−0.034	0.021	0.652
	Sex	0.022	0.015	1.405	−0.009	0.052	0.169
	APOE ε4 status	−0.022	0.020	−1.095	−0.060	0.017	0.276
	Time * Protocol	0.019	0.020	0.965	−0.020	0.059	0.337
	Timepoint * APOE ε4 status	0.065	0.024	2.735	0.018	0.111	0.007 **
	Protocol * APOE ε4 status	0.004	0.024	0.176	−0.042	0.050	0.861
	Time * Protocol * APOE ε4 status	−0.009	0.033	−0.274	−0.075	0.056	0.785
Right DTI-ALPS	Time	−0.029	0.012	−2.432	−0.053	−0.006	0.017 *
	Protocol	−0.027	0.012	−2.247	−0.051	−0.003	0.027 *
	Sex	0.006	0.010	0.552	−0.014	0.025	0.585
	APOE ε4 status	−0.046	0.015	−3.094	−0.076	−0.017	0.002 **
	Time * Protocol	0.022	0.017	1.278	−0.012	0.055	0.204
	Timepoint * APOE ε4 status	0.050	0.020	2.482	0.010	0.089	0.015 *
	Protocol * APOE ε4 status	0.024	0.020	1.208	−0.015	0.064	0.230
	Time * Protocol * APOE ε4 status	−0.015	0.028	−0.542	−0.071	0.040	0.589

*P* < 0.05 is denoted by a single asterisk (*) and *P* < 0.01 is denoted by a double asterisk (**). Results of the linear mixed-effects (LME) model examining the effects of Time, Protocol (iTBS, cTBS), and APOE ε4 Status (ε4 carriers and non-carriers), and their interactions on sham-controlled DTI-ALPS scores. Bold formatting highlights the interaction between APOE carrier status and ‘Timepoint’ (i.e., pre-/post-TBS).

**Figure 3:**
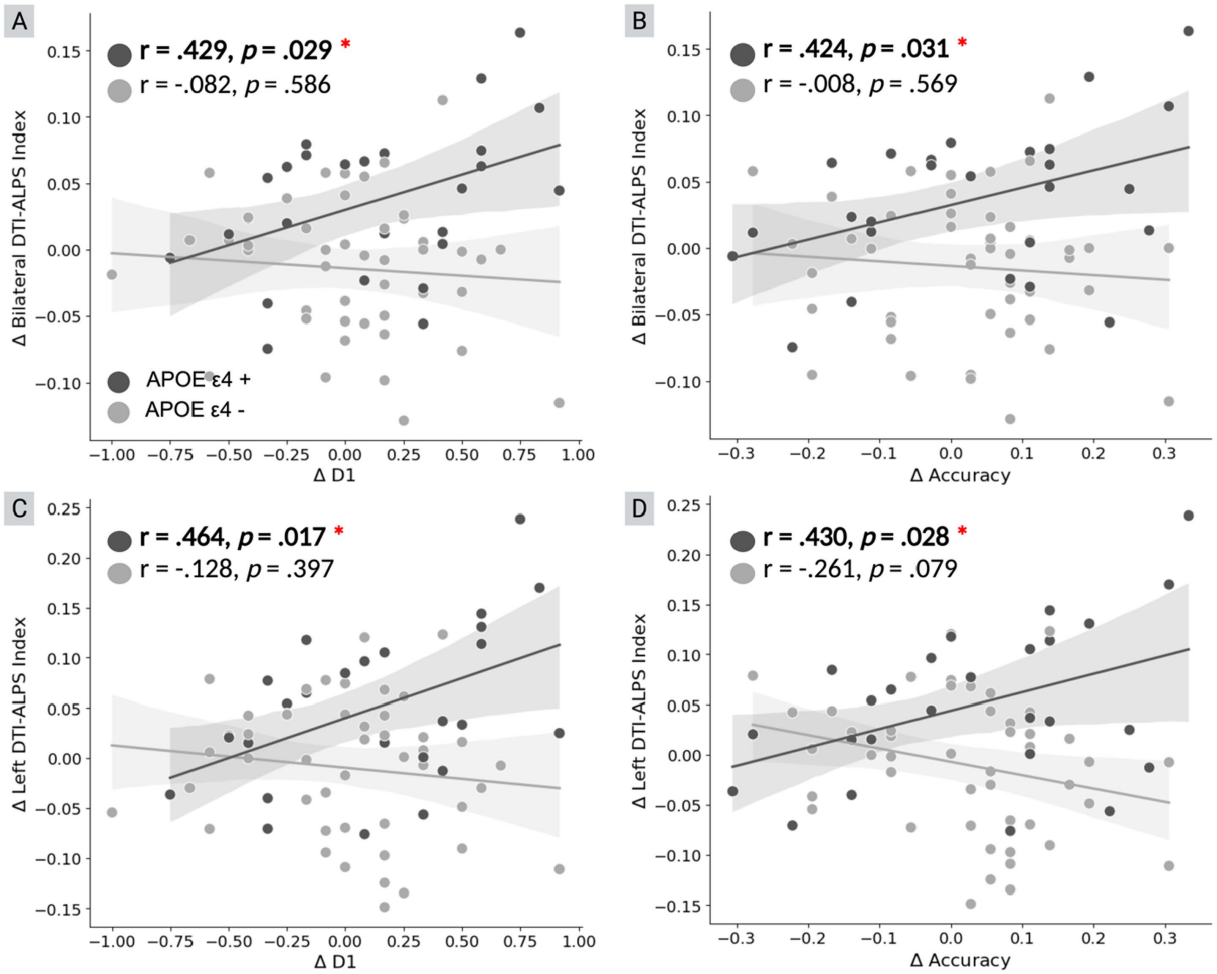
Association between *Δ* ALPS and FNAME performance by APOE ε4 status. Correlations between changes in DTI-ALPS index (Δ ALPS) and Face-Name Associative Memory Exam (FNAME) performance are shown, stratified by APOE ε4 status. **(A,C)** Correlations between Δ ALPS and Δ D1; **(B,D)** Correlations between Δ ALPS and Δ Accuracy. APOE ε4 carriers are represented by darker points, non-carriers by lighter points. Significant positive correlations were observed in APOE ε4 carriers only, with r and *p* values indicated in the plots.

Subsequent exploratory analyses assessed the relationship between changes in DTI-ALPS and associative memory as measured by the FNAME associative memory test following the TBS interventions. A greater increase in the DTI-ALPS was associated with larger improvements in FNAME scores, with this relationship being significant only in the APOE ε4 + group (*r* = 0.42–0.46, *p* < 0.05; Figure 3). The correlation analysis suggests that the observed changes in DTI-ALPS following active TBS may reflect glymphatic system plasticity, which is associated with cognitive improvements in APOE ε4 carriers.

## Discussion

This study is the first to evaluate changes in the DTI-ALPS index following theta-burst stimulation (TBS) in older adults with mild cognitive impairment (MCI), providing novel evidence of glymphatic plasticity. Notably, APOE ε4 carriers exhibited a significantly lower ALPS index at baseline, suggesting a potential glymphatic deficit that was selectively responsive to TBS. The observed increase in DTI-ALPS was observed regardless of whether iTBS or cTBS was applied, underscoring the capacity of the glymphatic system to be modulated by TMS interventions. Further, within this subgroup of APOE ε4 carriers, the increase in DTI-ALPS index correlated with memory improvements, supporting the functional relevance of glymphatic modulation in cognitive outcomes.

These novel findings in a human sample align with a series of recent studies of AD mouse models demonstrating that rTMS has potential to (1) enhance glymphatic function, (2) facilitate the clearance of AD pathology, and (3) improve cognitive function (Wu et al., 2022; Lin et al., 2021; Li et al., 2020). While the pronounced effect of APOE ε4 was unanticipated, it is consistent with its established contributions to the pathogenesis of AD. The APOE ε4 allele is the strongest genetic risk factor for sporadic AD, conferring a three-fold increase in risk for heterozygotes and a 15-fold increase for homozygotes (Farrer et al., 1997). Astrocytes, the brain’s primary source of APOE, are increasingly recognized as key mediators of APOE ε4-related dysfunction (Gee and Keller, 2005; Fernandez et al., 2019). APOE ε4 disruptions to glial function implicate glymphatic clearance, a glia-dependent process named to reflect the fundamental role of astrocytes in waste removal. Below, we explore the potential mechanisms by which TBS could enhance glymphatic function and highlight how these pathways may be influenced by the ε4 allele.

Importantly, our understanding of the glymphatic system remains in its early stages, and interpreting our results requires a degree of speculation in the absence of additional data. To ground our discussion, it is helpful to briefly outline the mechanisms underlying the most commonly described strategy for augmenting glymphatic function: sleep enhancement (van Hattem et al., 2024; Ma et al., 2024; Gottesman et al., 2024). Supporting the critical role of sleep in glymphatic clearance, studies consistently demonstrate that sleep deprivation impairs the removal of CSF tracers and Aβ proteins from the human brain (Vinje et al., 2023; Liu H. et al., 2023; Ooms et al., 2014; Eide et al., 2021; Shokri-Kojori et al., 2018). Beyond total sleep deprivation, mounting evidence highlights SWS as a key facilitator of this fluid exchange and clearance (Olsson et al., 2019; Ju et al., 2017; Varga et al., 2016). Several mechanisms explain the influence of SWS on glymphatic clearance. One is vasomotion, the rhythmic contraction and relaxation of blood vessels, which propels CSF through perivascular spaces to facilitate ISF exchange and waste clearance (van Veluw et al., 2020). Notably, imaging studies demonstrate a colocalization of neural oscillations and hemodynamic vasomotion during SWS (Fultz et al., 2019; Helakari et al., 2022). Relatedly, synchronized neuronal activity during sleep generates large amplitude ionic waves in the ISF, which may further promote CSF-ISF exchange and glymphatic clearance (Jiang-Xie et al., 2024).

Accordingly, sleep modulation may represent one plausible mechanism through which rTMS may exert influence on glymphatic function, with TMS-EEG studies providing insight into the immediate and delayed effects of TMS on neural dynamics during sleep. Huber and colleagues were among the first to demonstrate high-frequency (HF) rTMS applied to the motor cortex of healthy adults during wakefulness increased slow-wave activity during subsequent sleep periods (Huber et al., 2007). More recently, similar results were reported in a population of older adults with cognitive complaints, where HF-rTMS was associated with robust and widespread increases in slow-wave activity during subsequent sleep (Wilckens et al., 2024). This phenomenon aligns with use-dependent adaptations, where experimentally modulating synaptic strength during wakefulness bidirectionally affects slow-wave activity in subsequent sleep (Massimini et al., 2009). Authors of a review on the topic concluded that rTMS “can be used as a non-pharmacological means to controllably induce slow waves in the human cerebral cortex” (Massimini et al., 2009). In the context of APOE, a recent report from the Framingham Heart Study revealed that the typical SWS loss observed during aging is significantly accelerated in carriers of the ε4 allele (Himali et al., 2023). Additional evidence from a mouse model suggests that glymphatic function in ε4 carriers is particularly susceptible to the consequences of deficient sleep. Specifically, the study reported a feed-forward cycle of sleep disturbance, aberrant AQP4 polarization, and reduced glymphatic clearance of AD pathology that was only observed in the presence of the ε4 allele (Sadleir and Vassar, 2023). Considering these findings, it is plausible that the observed increase in DTI-ALPS may be attributable to rTMS-induced augmentation of SWS, which is a particularly vulnerable pathway in ε4 carriers (Himali et al., 2023; Sadleir and Vassar, 2023). However, in the absence of sleep data, this remains an untested hypothesis, and future studies incorporating sleep assessments will be essential to empirically evaluate this mechanism.

Relatedly, a growing body of evidence highlights the critical role of GABAergic interneurons in facilitating the heightened neural synchrony required for efficient glymphatic clearance during SWS (Chen et al., 2012). Knocking down GABAergic inhibition disrupts SWS, while optogenetic stimulation of cortical GABAergic neurons enhances slow-wave activity during deep sleep, further supporting their role in regulating SWS and glymphatic function (Thankachan et al., 2022; Chen et al., 2012). Inhibitory interneurons are particularly sensitive to TBS protocols, and their modulation is thought to be central to the after-effects of TBS (Li et al., 2019; Funke and Benali, 2011; Benali et al., 2011). Importantly, the GABAergic interneurons responsible for facilitating slow-wave activity are particularly vulnerable in APOE ε4 carriers, where their dysfunction leads to decreased synchronized neural activity (Najm et al., 2019). In aged mice, Gillespie et al. (2016) report that progressive disruption of interneuron-mediated slow-wave activity during sleep was specific to ε4 knock-in animals. This APOE ε4-dependent vulnerability in interneuron function may explain the baseline deficits observed in the DTI-ALP index in ε4 carriers, along with their enhanced response to our TBS intervention.

Beyond sleep and its associated slow-wave activity, animal model research suggests other potential mechanisms through which TBS may elicit an APOE4-dependent increase in DTI-ALPS, our proxy measure for glymphatic function. Several experiments in mouse models report an rTMS-induced increase in glymphatic function that is mediated by astrocytic remodeling (Lin et al., 2024; Lin et al., 2021; Wu et al., 2022). Specifically, these experiments report a suppression of astrocytic reactivity, which enhances the polarized distribution of AQP4 expression at the astrocytic endfeet (Lin et al., 2024; Lin et al., 2021; Wu et al., 2022). This polarized distribution is crucial for effective glymphatic clearance (Silva et al., 2021). Evidence from animal models demonstrates that a loss of AQP4 results in a significant reduction (about 70%) in glymphatic efflux, severely impairing the clearance of neurotoxic proteins such as Aβ and tau (Xu et al., 2015; Iliff et al., 2012; Harrison et al., 2020). Additionally, AQP4 dysfunction can occur when AQP4 channels are mislocalized away from astrocytic endfeet toward the cell soma, further compromising glymphatic function (Simon et al., 2022; Pedersen et al., 2023). Post-mortem studies demonstrate that AQP4 mislocalization is strongly linked to Alzheimer’s disease (AD) pathology (Zeppenfeld et al., 2017).

AQP4 mislocalization is also closely associated with astrocytic reactivity, a process that naturally occurs with aging or in response to stressors such as injury, neurodegeneration, or infection (Kress et al., 2014; Duncombe et al., 2017). The functional and morphological changes comprising astrocytic reactivity can be broadly classified as resembling an A1 or A2 phenotype. The neurotoxic A1 phenotype of astrocytes is associated with neuroinflammation, tissue damage, and acceleration of disease processes, whereas the A2 phenotype is generally considered protective, promoting repair and recovery (Fan and Huo, 2021). Recent evidence suggests that AQP4 mislocalization is tightly coupled to the A1 phenotype, which can be rescued by interventions that promote a shift to the A2 phenotype (Feng et al., 2023). Notably, APOE4 appears to promote astrocytes to adopt the A1 phenotype, while deleting APOE4 reduces harmful astrogliosis and restores AQP4 polarization (Wang et al., 2021; Koutsodendris et al., 2023). This is supported by human studies indicating that the relationship between Aβ and GFAP (a marker of A1 reactivity) is moderated by APOE4 (Snellman et al., 2023; Liddelow et al., 2017). Beyond studies directly demonstrating that TMS enhances AQP4 polarization to improve glymphatic function in AD models (Lin et al., 2024; Lin et al., 2021; Wu et al., 2022), additional evidence from murine stroke models suggests that TMS can also induce astrocytes to shift from an A1 to an A2 phenotype (Zong et al., 2020; Hong et al., 2020; Zou et al., 2024; Wang et al., 2025; Hu et al., 2023). A recent meta-analysis of rTMS clinical trials across neuropsychiatric disorders corroborates this effect, reporting that rTMS interventions significantly decrease inflammatory cytokines like tumor necrosis factor alpha (Asadizeidabadi et al., 2025). Collectively, these findings offer a plausible mechanistic explanation for the baseline deficits observed in APOE4 carriers and their selective responsiveness to TBS. However, this remains speculative in the absence of supporting human data, and future studies incorporating astrocytic biomarkers, such as plasma or CSF GFAP, will be essential to empirically evaluate astrocytic reactivity as a potential mediator of the observed effects.

Another experiment providing direct evidence that TBS enhances glymphatic function in an AD mouse model highlights a distinct mechanism. Specifically, this study reports that TBS upregulates vascular endothelial growth factor-C (VEGF-C), resulting in the dilation of meningeal lymphatic vessels (mLVs) (Li et al., 2020). mLVs exist downstream of ISF-CSF fluid exchange occurring at astrocytic endfeet to drain waste out of the CNS, and mLV dysfunction ultimately impairs this clearance (Guo et al., 2023). Further, despite existing downstream, mLVs appear to exert reciprocal influence on glymphatic function, as their ablation has been shown to promote A1-like astrocytic reactivity and AQP4 mislocalization (Wang et al., 2019). Progressive mLV dysfunction, characterized by reduced vessel diameter and coverage, is a feature of aging that is exacerbated by the APOE ε4 allele (Da Mesquita et al., 2018; Mentis et al., 2021).

Consistent with the findings that TBS dilated mLVs via VEGF-C signaling, previous studies highlight the plasticity of mLVs and their sensitivity to VEGF (Antila et al., 2017; Da Mesquita et al., 2021). For example, in one AD mouse model, VEGF-C therapy rescued mLV dysfunction and associated gliosis, leading to enhanced clearance of AD pathology (Da Mesquita et al., 2021). Another experiment in an AD mouse model revealed similar effects, with heightened relevance for ε4 carriers, reporting that a VEGF-dependent pathway mediates apoE4-driven pathologies. Specifically, VEGF levels were reduced in APOE4 mice, and treatment with VEGF reversed ε4-driven cognitive deficits and AD pathology (Salomon-Zimri et al., 2016). Post-mortem analysis of brain tissue provides supporting evidence that VEGF gene family expression levels produce APOE ε4 specific associations with cognitive aging. Collectively, these findings suggest that VEGF-mediated mLV (dys)function is another plausible mechanism that may explain our APOE-specific findings. Notably, however, this remains largely speculative and future research in human samples will be required to evaluate VEGF dynamics and mLV integrity in vivo, using biofluid markers and emerging imaging techniques (Ringstad and Eide, 2024).

While our study offers valuable insights, there are notable limitations that should be considered when interpreting our findings. First, DTI-ALPS itself has inherent anatomical and methodological constraints that must be recognized. Specifically, it measures water diffusivity along perivascular spaces in the deep white matter and does not directly assess whole-brain glymphatic clearance (Ringstad, 2024; Wright et al., 2024; Taoka et al., 2024; Boyd et al., 2024). As such, it cannot capture the full complexity of glymphatic fluid dynamics, including the clearance of larger molecules such as Aβ or waste trafficking through meningeal lymphatics (Ringstad, 2024). Additional critiques center on its anatomical constraints, contending that ALPS-measured diffusivity changes, which are constrained to deep white matter, may only reflect localized ISF dynamics that do not generalize to broader whole-brain waste clearance (Haller et al., 2024). Nevertheless, a growing body of literature supports the utility of the ALPS index as a proxy measure with functional relevance for glymphatic activity. Notably, it correlates strongly with intrathecal contrast MRI, which is considered the current gold standard for evaluating glymphatic function (Zhang et al., 2021), and it aligns with clinical markers of glymphatic disruption, such as apnea-hypopnea indices in individuals with obstructive sleep apnea (Ghaderi et al., 2025; Lee et al., 2022). Therefore, while DTI-ALPS should be interpreted with caution and not viewed as a comprehensive measure of glymphatic clearance, it provides non-invasive, functionally relevant insight into localized fluid dynamics (Taoka et al., 2024). As both the imaging method and the broader field of glymphatic research continue to evolve, our findings should be interpreted with appropriate caution. Future studies that combine ALPS-derived diffusivity changes with complementary methodologies will be critical for validating and contextualizing its use.

Additionally, this work could have been strengthened by the integration of key data that is omitted from this preliminary work and the inclusion of a larger sample size. First, the absence of Alzheimer’s disease biomarkers limits the ability to directly link our findings with disease-specific neuropathology. Future integration of plasma/CSF biomarkers could address this limitation and also serve to substantiate our proxy measure of glymphatic function by examining whether changes in the DTI-ALPS index correspond with changes in Aβ levels. Second, the omission of sleep data constrains our ability to evaluate a potential mechanism underlying the TBS-induced DTI-ALPS changes and prevents us from accounting for sleep as a potential confounding factor in our analyses (Ma et al., 2024; Lee et al., 2022; Ghaderi et al., 2025). Lastly, although we documented neuropsychiatric history and conducted standardized neuropsychological assessments, broader clinical characterization was not obtained. The absence of information on metabolic or cardiovascular comorbidities poses a limitation, as these factors may also impact brain health and glymphatic function (Zhang et al., 2025; Yu et al., 2024; Tian et al., 2024). These comorbidities may also interact with APOE status in ways that could help contextualize the ε4-specific effects observed in this study (Kyrtsos and Baras, 2015; Shaaban et al., 2019; Nation et al., 2016; Kaufman et al., 2021). Lastly, the modest sample size—notably with an uneven distribution of ε4 carriers - constrains the overall interpretability of our findings and precludes subgroup analyses of different MCI subtypes, which could reveal differential effects across this heterogeneous population.

In addition to addressing the limitations detailed above, future research may also benefit from incorporating biofluid measures of GFAP, a measure of astrocytic reactivity, and VEGF, which has been linked to rTMS efficacy in clinical trials for depression (Fukuda et al., 2020; Elemery et al., 2022). These additions could further substantiate the DTI-ALPS index as a proxy for glymphatic function, elucidate the biological pathways involved, and provide deeper insight into how neuromodulatory interventions exert their effects. Additionally, although our statistical analyses did not reveal significant protocol-specific effects between cTBS and iTBS, future work should investigate whether other aspects of the broader TMS parameter space influence outcomes. It remains unclear whether variations to stimulation intensity, session number, pulse count, and/or cortical target selection might modify the effects observed in this sample. Moreover, while the FNAME served as a sensitive and targeted measure of associative memory (Rentz et al., 2011; Rubiño and Andrés, 2018), its use as the sole cognitive outcome limits the ability to draw broader conclusions about functional relevance. To better understand the cognitive impact of neuromodulation-related brain changes, future studies should include full neuropsychological batteries that evaluate a wider range of domains, including executive functioning, attention, and processing speed. Finally, the homogeneity of our sample, which was predominantly right-handed, highly educated, and female, may limit the generalizability of these findings. Replication in more diverse and representative populations will be essential to strengthen external validity and inform the broader applicability of rTMS interventions.

## Conclusion

We utilized DTI-ALPS as a proxy measure for the glymphatic system in older adults with MCI, assessing baseline function and its plasticity in response to TMS. Our results suggest that APOE e4 carriers exhibit a baseline glymphatic deficit that was selectively responsive to iTBS and cTBS interventions compared to sham stimulation. Supporting the functional relevance of glymphatic plasticity, TBS-induced increases in the ALPS index were associated with improved memory performance within this subgroup of e4 carriers. This work highlights the capacity of TMS to modulate glymphatic function, suggesting that this pathway may contribute to its therapeutic effects. While we propose several plausible mechanisms underlying the APOE-specific effects observed, further research is needed to directly investigate these potential pathways and their therapeutic implications. Importantly, this work remains exploratory, requiring replication in larger and more diverse samples, as well as confirmation using complementary methodologies. Future studies should integrate fluid biomarkers such as plasma Aβ and GFAP to further validate the DTI-ALPS index as a proxy for glymphatic function and to assess astrocytic reactivity as a potential mechanism underlying the APOE-specific effects observed in this preliminary work.

## Data Availability

The original contributions presented in the study are included in the article/Supplementary material, further inquiries can be directed to the corresponding author.
