# Supplementary material for: The glymphatic system as a therapeutic target: TMS-induced modulation in older adults
Source: Front Aging Neurosci. 2025 Jul 17;17:1597311. doi: 10.3389/fnagi.2025.1597311 (PMC12311639; doi:10.3389/fnagi.2025.1597311)
Supplement: Supplementary file 1 [file Data_Sheet_1.docx]

**Supplementary Materials**

**S1 Participants**

The dataset consisted of 36 right-handed individuals with mild cognitive impairment (MCI; age: 66.1 ± 7.45 years old; females: 28, education: 16.375 ± 2.21 years). Participants were recruited from the Tucson, Arizona area through memory clinics affiliated with the University of Arizona, as well as through community advertisements and local outreach efforts. The recruitment period lasted from November 2020 to November 2023. The study’s exclusion criteria encompassed the following factors: (i) individuals with diagnoses of brain injury, cardiovascular disease, or other neurological diseases such as dementia or Parkinson’s disease; (ii) untreated psychiatric symptoms meeting DSM-IV criteria (e.g., depression, anxiety, substance use disorder); and (iii) any existing contraindications for TMS and MRI that may pose safety risks. All study procedures were reviewed and approved by the University’s Institutional Review Board. Written informed consent was obtained from each participant prior to participation, in accordance with the Declaration of Helsinki. Participants were compensated for their time. Upon completion of a safety screening questionnaire, participants were deemed eligible to undergo both MRI and TMS procedures. All participants were free from any known neurological or psychiatric conditions that could confound study outcomes or increase risk, including a history of seizures, Parkinson’s disease, stroke, dementia, or major depressive disorder.

**S2 Single-Pulse Transcranial Magnetic Stimulation**

We used single-pulse TMS with the ‘CB60’ figure-of-eight coil (MagVenture, Denmark) to estimate resting motor threshold (RMT) at the beginning of each TBS condition. First, the participant’s structural T1 MRI data was uploaded into a 3D Neuronavigation platform and carefully co-registered to landmarks on the participant’s head using an infrared-based frameless stereotactic system (Polaris System, Localite Version 3.0.41). To identify the motor hotspot corresponding with the right abductor pollicis brevis (APB) thumb muscle, the motor ‘hand knob’ area was visually identified on the left precentral gyrus from each participant’s structural T1 MRI data. The motor hotspot was subsequently refined based on observed motor-evoked potentials (MEPs) from the APB thumb muscle. The optimal motor hotspot was recorded with the neuronavigation system, which enabled precise coil positioning throughout the single-pulse TMS session. The RMT was estimated with the TMS Motor Threshold Assessment Tool 2.0 (http://www.clinicalresearcher.org/software.htm) that uses maximum-likelihood parameter estimation by sequential testing strategy without the need for a priori information. The estimated RMT was further verified by eliciting at least three MEPs of 50-μV peak-to-peak amplitude of the right APB thumb muscle measured with an electromyography machine in six consecutive stimulations on the hotspot of the left primary motor cortex. Once the RMT was determined, the stimulation intensity for TBS was set to 70% of individual RMT. RMTs were measured at the first three sessions for each block, and the averaged stimulation intensity was used for subsequent TBS sessions in that block.

**S3 MRI Acquisition**

MRI data were acquired by MAGNETOM® Skyra 3 Tesla MRI scanner (Siemens Medical Systems, Erlangen, Germany) with a 32-channel receiver head coil. Foam pads were applied to prevent head motion. Diffusion-weight MRI (single-shot parallel and multi-band dual-spin-echo EPI pulse sequence) parameters with FoV= 256 mm; in-plane matrix size = 128 × 128; in-plane acceleration factor = 2; multi-band factor = 2; TE = 119 msec; TR = 3700 msec; slice thickness = 2 mm; voxel size = 2 mm^3^; b = 0, 1000, 2000, and 3000 s/mm^2^ as three shell acquisitions for further high angular resolution diffusion imaging (HARDI) approach; number of diffusion-encoding directions = 60; scan time = 9 minutes.

**S4 Identification of Stimulation Sites with Diffusion Tensor Image**

For the creation of a voxel-wise probabilities tractography map, we employed FSL’s Bedpostx and Probtrackx tools. The diffusion tensor was fitted using the Bayesian Estimation of Diffusion Parameters Obtained using Sampling Techniques (BEDPOSTX) method (Jeurissen et al.), which models diffusion signals as ‘ball and stick’ components to generate fiber orientation distribution within each voxel. We used probabilistic tractography probtrackx (FSL) to reconstruct the distribution probability of tracks. It can build the distributions on diffusion parameters at each voxel and repeatedly sample voxel-to-voxel propagation of streamlines until stopping criteria are met (Behrens et al., 2003, Behrens et al., 2007). We utilized Probtrackx from FSL for the reconstruction of track distribution probabilities, providing maps that reflect the likelihood of fiber presence within a voxel. This method accounts for crossing-fiber pathways and builds distributions of diffusion parameters at each voxel, repeatedly sampling voxel-to-voxel streamline propagation until predetermined stopping criteria are met. Using this approach, we computed the number of tracts extending from the seed mask (hippocampal body) to the grey matter cortical surface, normalized by the total number of tracts from the seed ROI, thereby generating a probability map from the hippocampal body to the rest of the brain. For this process, we initiated 50,000 streamlines per voxel in the seed mask and produced a probability distribution until they terminated in voxels within the grey matter cortical areas, with a step length of 0.5 mm, curvature threshold of 0.2 and a ‘loop check’ to exclude tracks that double back on themselves. Additional constrains were applied to the probability map by using the parietal lobe masks. The constrained probability maps were further clustered using the t-stats map with p = 0.9. The clusters that survived were then ranked by the center of volume (COV). The cluster with the highest COV within the mask was selected as the stimulation site.

**S5 Face-name Associative Memory Task**

All participants performed a face-name associative memory task consisting of the encoding and retrieval phases. During the encoding phase, participants were instructed to memorize the face-name pairs and make a subjective decision on how well the name fit the corresponding face on a scale from 1 (poor match) to 4 (good match), a strategy designed to augment associative encoding (Carr et al., 2017). There were 24 face-name pairs during the encoding phase. Following the encoding phase, there was a 5-minute break. During the retrieval phase, participants were instructed to decide whether the presented face-name pairs were "Correct” (i.e., the face-name pairs were presented during the encoding phase), “Incorrect” (i.e., the faces and names were presented during the encoding phase but a wrong combination) or “New” (i.e., neither the names nor the faces were presented during the encoding phase). In total, there were 36 pairs consisting of 12 “Correct”, 12 “Incorrect”, and 12 “New” pairs during the retrieval phase (each lasting 5.5 seconds). Faces were adopted from the Chicago face database, and were randomly and evenly distributed according to race, gender, and age into 6 different versions. Participants were administered different versions of the face-name task before and after each TBS condition, in a randomized order for all participants. Several behavioral outcome measures were derived from the Face-name associative memory task. First, we computed absolute accuracy by tallying the total number of accurate responses out of all the trials. Second, following Carr et al., 2017 (Carr et al., 2017), we categorized the recognition results into nine groups (3 x 3) based on 3 types of face-name pairs and 3 types of responses. For example, the Hit rate represented the rate of accurately responding “Correct” to “Correct” pairs (CC), while the False alarm rate was defined as the rate of wrongly responding “Incorrect” to “Correct” pairs (CI). Additionally, a sensitivity measure (d1) that quantified the ability to distinguish between signal and noise, was defined by *Z*(Hite rate) – *Z*(False alarm rate). These behavioral metrics were computed using the same methodology for all three TBS protocols.

**Supplementary Table 1. Descriptive Statistics of DTI-ALPS Index by APOE ε4 Status, Stimulation Protocol, and Timepoint**

| **Outcomes** | **APOE ε4 Status** | **Protocol** | **Timepoint** | **Mean** | **SD** |
| --- | --- | --- | --- | --- | --- |
| Bilateral DTI-ALPS Index | APOE ε4- | iTBS | pre | 0.015 | 0.042 |
|  |  |  | post | -0.009 | 0.036 |
|  |  | cTBS | pre | -0.001 | 0.047 |
|  |  |  | post | -0.005 | 0.044 |
|  | APOE ε4+ | iTBS | pre | -0.019 | 0.042 |
|  |  |  | post | 0.014 | 0.035 |
|  |  | cTBS | pre | -0.021 | 0.047 |
|  |  |  | post | 0.020 | 0.043 |
| Left DTI-ALPS Index | APOE ε4- | iTBS | pre | 0.005 | 0.057 |
|  |  |  | post | -0.014 | 0.052 |
|  |  | cTBS | pre | -0.001 | 0.064 |
|  |  |  | post | -0.001 | 0.053 |
|  | APOE ε4+ | iTBS | pre | -0.016 | 0.050 |
|  |  |  | post | 0.028 | 0.058 |
|  |  | cTBS | pre | -0.019 | 0.072 |
|  |  |  | post | 0.036 | 0.049 |
| Right DTI-ALPS Index | APOE ε4- | iTBS | pre | 0.025 | 0.049 |
|  |  |  | post | -0.004 | 0.041 |
|  |  | cTBS | pre | -0.002 | 0.044 |
|  |  |  | post | -0.009 | 0.048 |
|  | APOE ε4+ | iTBS | pre | -0.021 | 0.046 |
|  |  |  | post | -0.001 | 0.027 |
|  |  | cTBS | pre | -0.024 | 0.031 |
|  |  |  | post | 0.003 | 0.045 |

References

BEHRENS, T. E., BERG, H. J., JBABDI, S., RUSHWORTH, M. F. & WOOLRICH, M. W. 2007. Probabilistic diffusion tractography with multiple fibre orientations: What can we gain? *Neuroimage,* 34**,** 144–55.

BEHRENS, T. E., WOOLRICH, M. W., JENKINSON, M., JOHANSEN-BERG, H., NUNES, R. G., CLARE, S., MATTHEWS, P. M., BRADY, J. M. & SMITH, S. M. 2003. Characterization and propagation of uncertainty in diffusion-weighted MR imaging. *Magn Reson Med,* 50**,** 1077–88.

CARR, V. A., BERNSTEIN, J. D., FAVILA, S. E., RUTT, B. K., KERCHNER, G. A. & WAGNER, A. D. 2017. Individual differences in associative memory among older adults explained by hippocampal subfield structure and function. *Proc Natl Acad Sci U S A,* 114**,** 12075–12080.

JEURISSEN, B., LEEMANS, A., TOURNIER, J. D., JONES, D. K. & SIJBERS, J. 2013. Investigating the prevalence of complex fiber configurations in white matter tissue with diffusion magnetic resonance imaging. *Hum Brain Mapp,* 34**,** 2747–66.
